# Current status of FAP-directed cancer theranostics: a bibliometric analysis

**DOI:** 10.52601/bpr.2024.240022

**Published:** 2024-12-31

**Authors:** Dan Ruan, Simin Wu, Xuehua Lin, Liang Zhao, Jiayu Cai, Weizhi Xu, Yizhen Pang, Qiang Xie, Xiaobo Qu, Haojun Chen

**Affiliations:** 1 Department of Nuclear Medicine and Minnan PET Center, Xiamen Key Laboratory of Development and Translation of Radiopharmaceuticals, the First Affiliated Hospital of Xiamen University, School of Medicine, Xiamen University, Xiamen 361102, Fujian, China; 2 National Institute for Data Science in Health and Medicine, Department of Electronic Science, Intelligent Medical Imaging R & D Center, Fujian Provincial Key Laboratory of Plasma and Magnetic Resonance, Xiamen University, Xiamen 361102, Fujian, China; 3 Department of Cardiology, the First Affiliated Hospital of Xiamen University, School of Medicine, Xiamen University, Xiamen 361102, Fujian, China; 4 Xiamen Key Laboratory of Rare Earth Photoelectric Functional Materials, Xiamen Institute of Rare Earth Materials, Haixi Institute, Chinese Academy of Sciences, Xiamen 361021, Fujian, China

**Keywords:** Cancer, FAPI, Fibroblast activation protein, PET/CT, Radionuclide, Bibliometric analysis

## Abstract

Fibroblast activation protein (FAP) is a key molecule in the field of oncology, with significant impacts on tumor diagnosis and treatment. Importantly, it has paved the way for the development of radiotracers for quinoline-based FAP inhibitors (FAPIs), which are currently among the most promising radiotracers for PET imaging in cancer. We performed a bibliometric analysis of scientific publications related to FAP and FAPI-based radiotracers, which included the quantification and visualization of current research trends and prospects based on various bibliometric indicators. In our survey of FAP-related studies in the Web of Science Core Collection databases, R and VOSviewer were used for visualization and bibliometric analyses based on country, institute, author, journal, and keywords. We also examined the methodology, radionuclide type, imaging instruments, and major diseases associated with studies on FAPI-based radiotracers. The results revealed 2,664 FAP-related publications from 1992 to the present. Germany, the USA, and China dominated paper publications, multinational collaborations, and societal impacts on FAP research. Southwest Medical University was the most productive institute, while Haberkorn Uwe authored the most cited papers and the highest H-index. The *European Journal of Nuclear Medicine and Molecular Imaging* and the *Journal of Nuclear Medicine* were the most influential periodicals. Keywords "FAP", "^68^Ga-FAPI", and "PET/CT" emerged as the most significant in this field. This study may help elucidate current research trends, hotspots, and directions for future research.

## INTRODUCTION

Fibroblast activation protein-α (FAP) is the cell-surface antigen expressed by cancer-associated fibroblasts (CAFs) in the tumor microenvironment (TME). Initially, this antigen was detected in most human astrocytomas, sarcomas, and some melanomas using the monoclonal antibody F19 (Rettig *et al.* 1986, 1988). Subsequently, FAP was also identified in the reactive stroma of epithelial carcinomas, granulation tissue during wound healing, and malignant cells of bone tissue (Garin-Chesa *et al.* 1990; Scanlan *et al.* 1994). In normal adult tissues, FAP is generally absent (Scanlan *et al.* 1994). Therefore, FAP has shown promise as a prognostic biomarker in a variety of malignancies. For instance, high FAP expression has been associated with poor outcomes in pancreatic, gastric, and oral squamous cell carcinomas (Hu *et al.* 2017; Shi *et al.* 2012; Wang *et al.* 2014). In inflammatory diseases, the reduced circulating FAP within the first 5 days is associated with increased mortality in acute ST-elevation myocardial infarction (Tillmanns *et al.* 2017). FAP is highly expressed in arthritic joints, and its deficiency can positively impact cartilage destruction in cases of inflammatory destructive arthritis (Laverman *et al.* 2015; Waldele *et al.* 2015). In radioimmunoimaging, the severity of joint inflammation can be visualized using anti-FAP antibodies. For instance, SPECT and PET imaging have been used to quantify the uptake of^ 111^In- and ^89^Zr-labeled anti-FAP antibodies 28H1 in joints (Laverman *et al.* 2015; van der Geest *et al.* 2018). In tumors, FAP expression is detected using near-infrared (NIR) fluorescent probes and quinoline-based FAP-targeted radiotracers (^68^Ga/^18^F-labeled FAP inhibitor [FAPIs]), which have shown intense tumor uptake and favorable image contrast capabilities in various tumors (Li *et al.* 2012; Lindner *et al.* 2018; Xing *et al.* 2018).

Because cancerous stroma contributes to cancer recurrence and treatment resistance, many initial therapeutic approaches directly targeting cancer cells were inadequate for eliminating tumors. Targeting stromal cells in the TME may effectively treat solid tumors of epithelial origin. This involves depleting or destroying all FAP-expressing cells, including stromal and cancer cells, which can indirectly inhibit tumor cell proliferation, increase collagen accumulation, decrease tumor vascular growth, and ultimately lead to rapid tumor death (Santos *et al.* 2009). Hence, tumor growth and metastasis can be effectively inhibited using FAP antibodies, FAP-targeted radiopharmaceuticals, or silencing FAP expression (Cheng *et al.* 2002; Fendler *et al.* 2022; Liu *et al.* 2022; Wang *et al.* 2014). Combining FAP-targeted vaccines or antibodies with chemotherapeutic agents or radiotherapy can enhance antitumor efficacy by modulating the TME (Labiano *et al.* 2021; Xia *et al.* 2016). These findings suggest that FAP may serve as a target for disrupting FAP-driven tumor progression and eradicating malignant tumors.

A substantial volume of clinical and basic research has been published in the field of FAP-directed theranostics. However, very few studies have characterized the trends in FAP-related research and how they relate to the advancement of FAPI-based radiotracers. To bridge this gap, our study conducted a comprehensive bibliometric analysis of scientific publications on FAP and FAPI-based radiotracers. Through this analysis, we quantified and visually described important and reliable scientific publications using various bibliometric indicators, elucidating current research hotspots, trends, and relevant research directions.

## RESULTS

### Overview of retrieved data

Between 1992 and 2024, a total of 2,664 publications were retrieved, generating 72,817 references and involving 12,611 authors (supplementary Table S1). Single-author publications numbered 56, while those with multiple authors had an average of eight coauthors per paper. International co-authorship constituted 22.2% of all publications.

### Countries' productivity and collaboration

**Figure 1:**
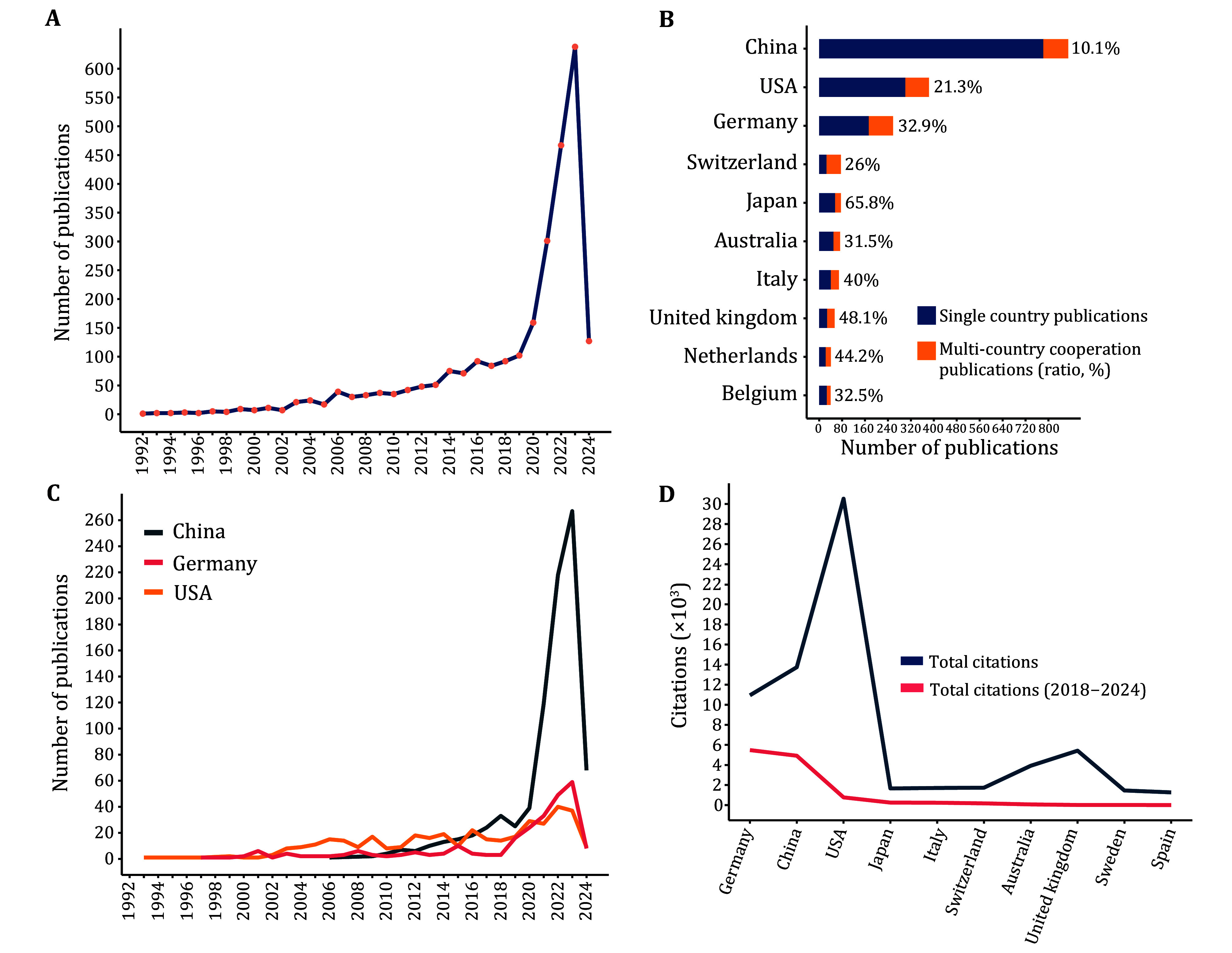
Global trends in FAP-based research from 1993 to 2024 (**A**). Country distribution and co-authorship of FAP-related publications (**B**), trends in the number of publications per year (**C**), and citations (**D**)

**Figure 2:**
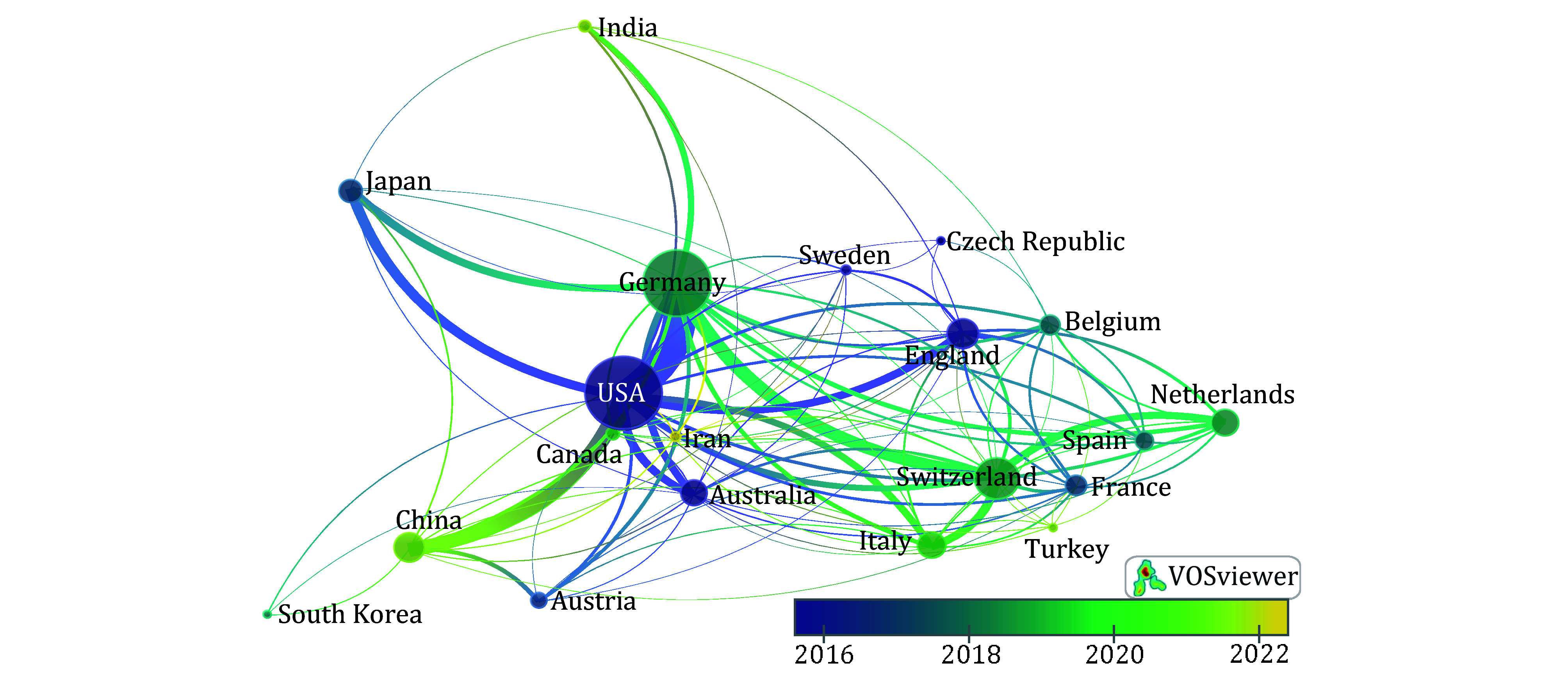
Overlay visualization on the cooperation of major countries. The connection strength between countries is determined by the number of co-authored articles. Node size and line thickness indicate the intensity of cooperation between countries; node color reflects the average literature publication time for each country. The specific parameters set for this analysis are: Type of analysis = Co-authorship; Unit of analysis = Countries; Minimum number of documents per country = 30; Weights = Total link strength; Normalization method = Association strength

From 1992 to 2018, there was a gradual increase in the number of publications; however, starting in 2019, there was a rapid surge (Fig. 1A). China led with 883 articles, of which 10.1% (89/883) featured multinational co-authorship (Fig. 1B). The USA followed with 303 articles, with 21.3% (82/385) involving multinational collaboration. Germany stood out with the highest percentage of multinational co-authorship (32.9%, 85/258). In 2021, China and Germany surpassed the USA in the number of annual publications, with a rising trend (Fig. 1C). This shift reflected early US research on FAP-targeted immunotherapy and FAP expression in tumors, while German studies from 2018 focused on radiolabeled FAPIs for PET imaging, experiencing explosive growth. US articles received the most global citations, followed by Chinese articles (Fig. 1D). However, due to the explosive growth of radiolabeled FAPIs, Germany had the highest number of global citations since 2018. Strong international links were noted among Germany, the USA, China, Japan, and Switzerland (Fig. 2). The earliest FAP-related studies originated in the USA (average publication year: <2016), followed by Germany and Switzerland (2018–2020), and China (2021).

### Most relevant institutions, journals, and authors

**Figure 3:**
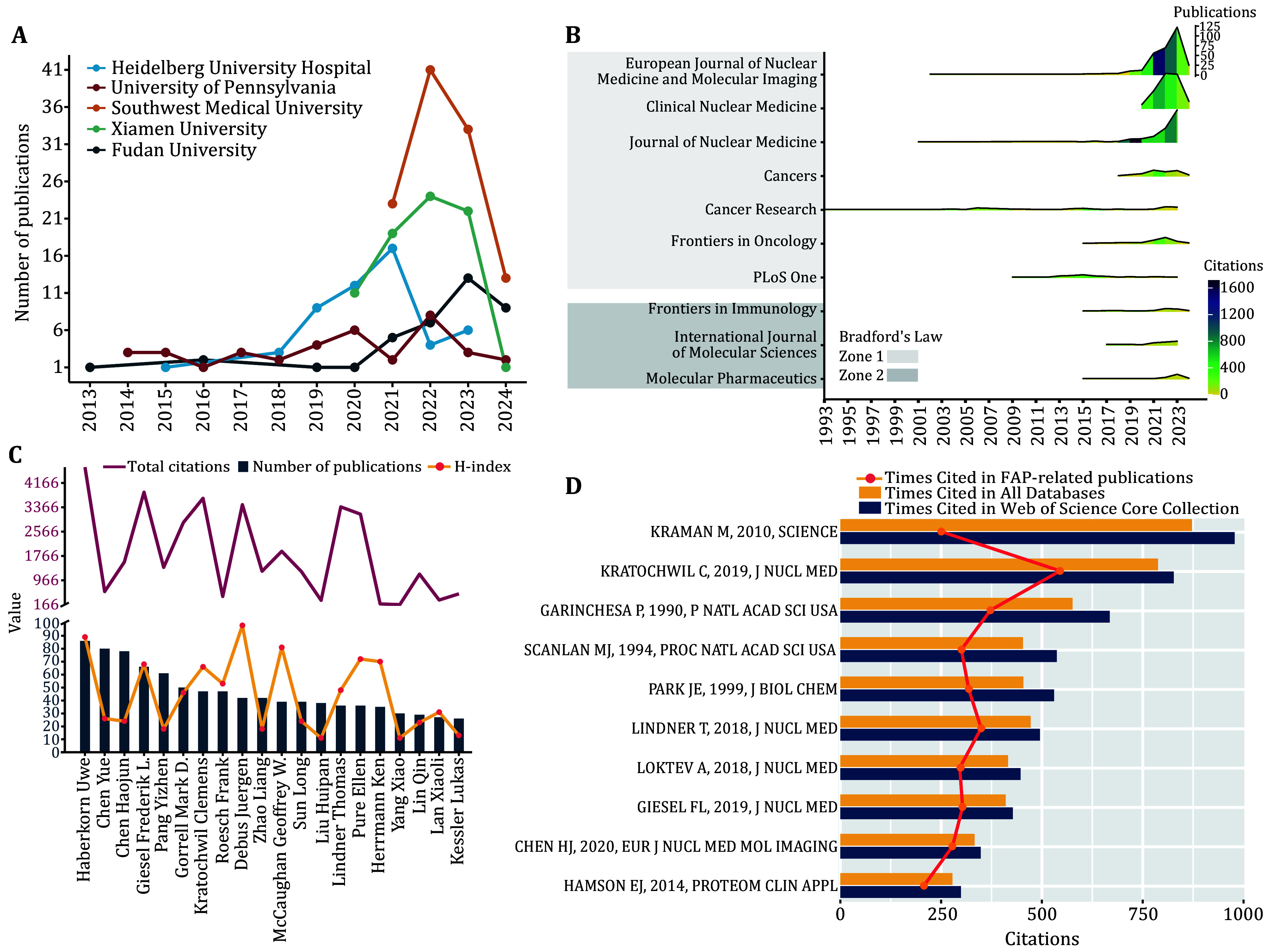
Characteristics of research on FAP: analysis of top units, authors, and studies. Cumulative increase in number of articles per research center over time (**A**). The journal classification is based on Bradford's Law. The height of the ridges represents the number of publications, while the depth of the ridge color indicates the number of citations. *Journal of Nuclear Medicine* (JNM) saw a peak in citations from 2019 to 2020, and *European Journal of Nuclear Medicine and Molecular Imaging* (EJNMMI) experienced a peak from 2021 to 2022. The number of publications for EJNMMI reached a high of 122 in 2023, for *Clinical Nuclear Medicine* (CNM) it peaked at 90 in 2022, and for JNM it peaked at 83 in 2023 (**B**). Number of publications, citations, and H-index of the relevant authors (**C**). Citations of related literature (**D**)

The top five institutions by publication count were Southwest Medical University, Xiamen University (The First Affiliated Hospital of Xiamen University), Heidelberg University Hospital, Fudan University, and the University of Pennsylvania (Fig. 3A). Since 2018, the number of articles from Heidelberg University Hospital has grown rapidly, followed by rapid growth in publications from Xiamen University starting in 2020, and a surge from Southwest Medical University since 2021. FAP-related studies are concentrated in core journals, particularly the *European Journal of Nuclear Medicine and Molecular Imaging* (EJNMMI), the *Clinical Nuclear Medicine* (CNM), and the *Journal of Nuclear Medicine* (JNM), classified as Zone 1 by Bradford’s Law (Bradford 1985) (Fig. 3B). EJNMMI and CNM had more articles but fewer total citations than JNM (supplementary Table S2). EJNMMI’s total citations have surged since 2021, while JNM saw a notable increase since 2019 (Fig. 3B).

**Figure 4:**
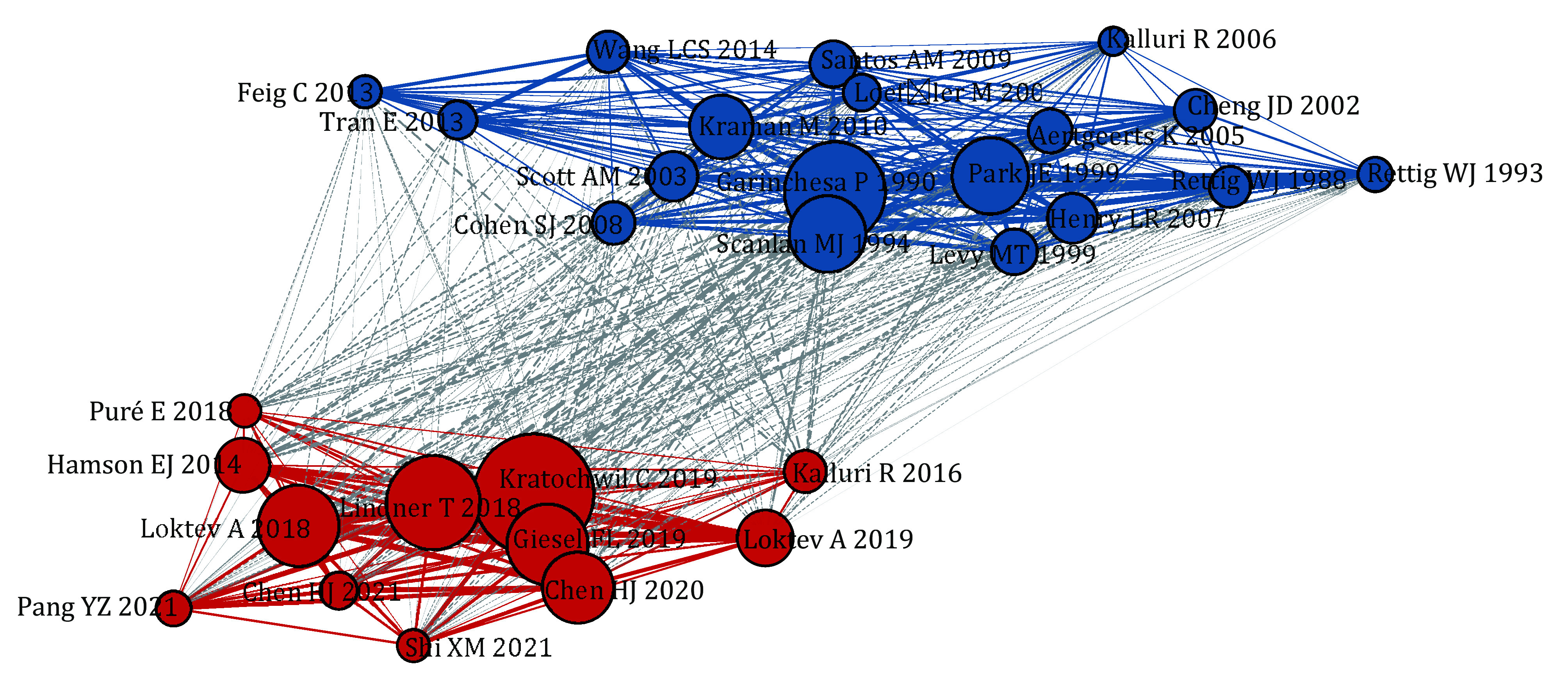
Co-citation network of the most relevant literature on FAP. The blue citations represent the early discovery of FAP and the exploration of its structure and function. Early research using the monoclonal antibody F19 identified FAP expression in the reactive stroma of primary and metastatic epithelial tumors (Garin-Chesa *et al*. 1990). Subsequent studies revealed the molecular structure of FAP and highlighted differences in its expression patterns among tumor fibroblasts, sarcomas, and fibroblasts involved in wound healing (Scanlan *et al*. 1994). Later, FAP enzymatic activity was detected in human cancer tissues (Park *et al*. 1999). It was also discovered that FAP-expressing cells play an immunosuppressive role within the tumor microenvironment (Kraman *et al*. 2010). The red citations represent the development and clinical application of FAPI-based tracers. Based on reviews of FAP's expression in malignancies, its role as a biomarker, and its potential as a therapeutic target (Hamson *et al*. 2014, Kalluri 2016, Puré and Blomberg 2018), the research team from Heidelberg University Hospital primarily drove the development and initial clinical applications of FAPI-based tracers (Lindner *et al*. 2018, Kratochwil *et al*. 2019)

Haberkorn Uwe led with 86 publications, holding the highest H-index (89) and the highest number of total citations (4,684). Chen Yue and Chen Haojun followed with 80 and 78 publications and H-indexes of 26 and 24, respectively (Fig. 3C). Among the top ten influential articles, five were FAPI-based tracer studies authored by German and Chinese researchers, published in JNM and EJNMMI, respectively. The remaining articles focused on the expression of FAP in cancer stroma and FAP-targeted immunotherapy (Fig. 3E and supplementary Table S3). The co-citation network of FAP research shows a dichotomy: one group is centered on FAPI tracers, while the other focuses on non-radioactive FAP-targeted therapy and the expression of FAP in the tumor microenvironment (Fig. 4).

**Figure 5:**
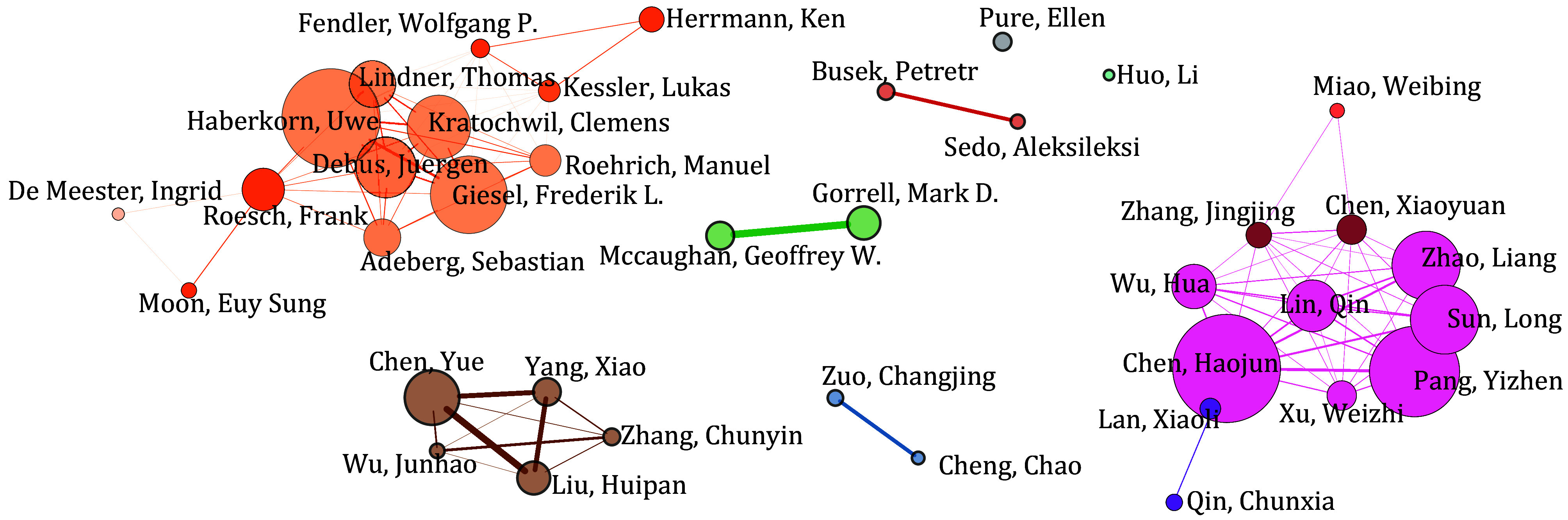
Collaborative network map of key authors

Researchers at Heidelberg University Hospital collaborated with the University of Duisburg-Essen via Fendler WP and Kessler L, and with Johannes Gutenberg University Mainz and the University of Antwerp via Roesch Frank. Another collaborative network of multicenter researchers has been established among Huazhong University of Science and Technology, Xiamen University, the National University of Singapore, and Fujian Medical University through the efforts of Lan Xiaoli, Chen Haojun, Zhang Jingjing, Chen Xiaoyuan, and Miao Weibing (Fig. 5).

### Keywords analysis

**Figure 6:**
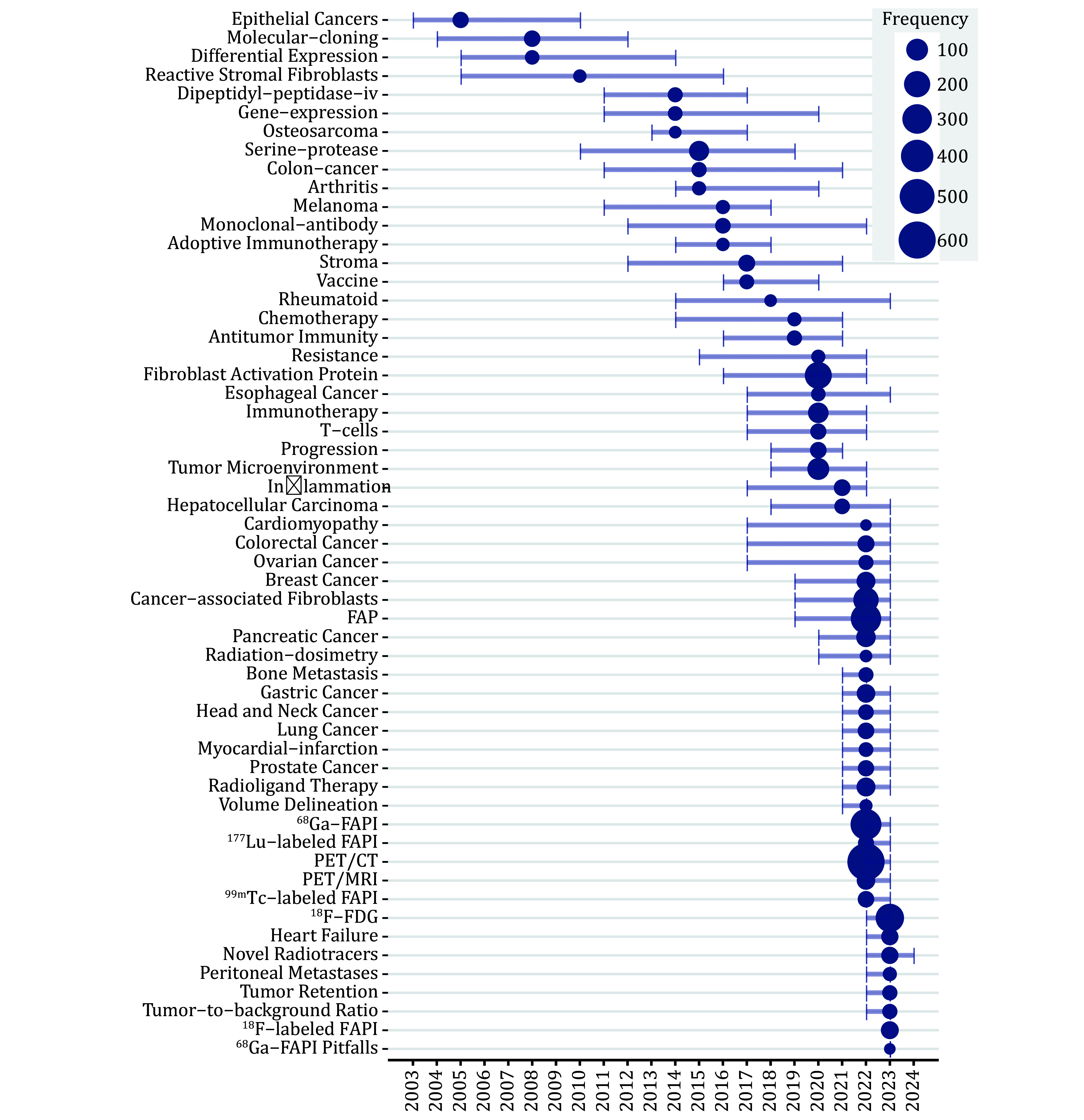
Annual distribution of keywords. Larger dots indicate higher frequencies

From 2003 to 2019, keywords shifted from epithelial cancer, molecular cloning, gene expression, and serine-protease to stroma, vaccine, rheumatoid, chemotherapy, and antitumor immunity, showcasing the evolution of FAP-related research (Fig. 6). This evolution began with the discovery of the F19 monoclonal antibody recognizing FAP in various epithelial carcinomas, leading to immunotherapy and immune-combination chemotherapy. Post-2019, "FAP", "^68^Ga-FAPI", and "PET/CT" became predominant, reflecting extensive research on tumor diagnosis using PET/CT since the development of FAPI-based radiotracers in 2018. Besides various cancers, inflammation and cardiovascular disease were common keywords. PET/MRI gained predominance from 2022, mirroring the increased use of PET/MRI in disease diagnosis. Starting in 2022, terms like radiation-dosimetry, radionuclide therapy, volume delineation, tumor retention, and tumor-to-background ratio became popular, highlighting the growing interest in FAP-targeted radioligand therapy.

### Application of radiolabeled FAPIs

**Figure 7:**
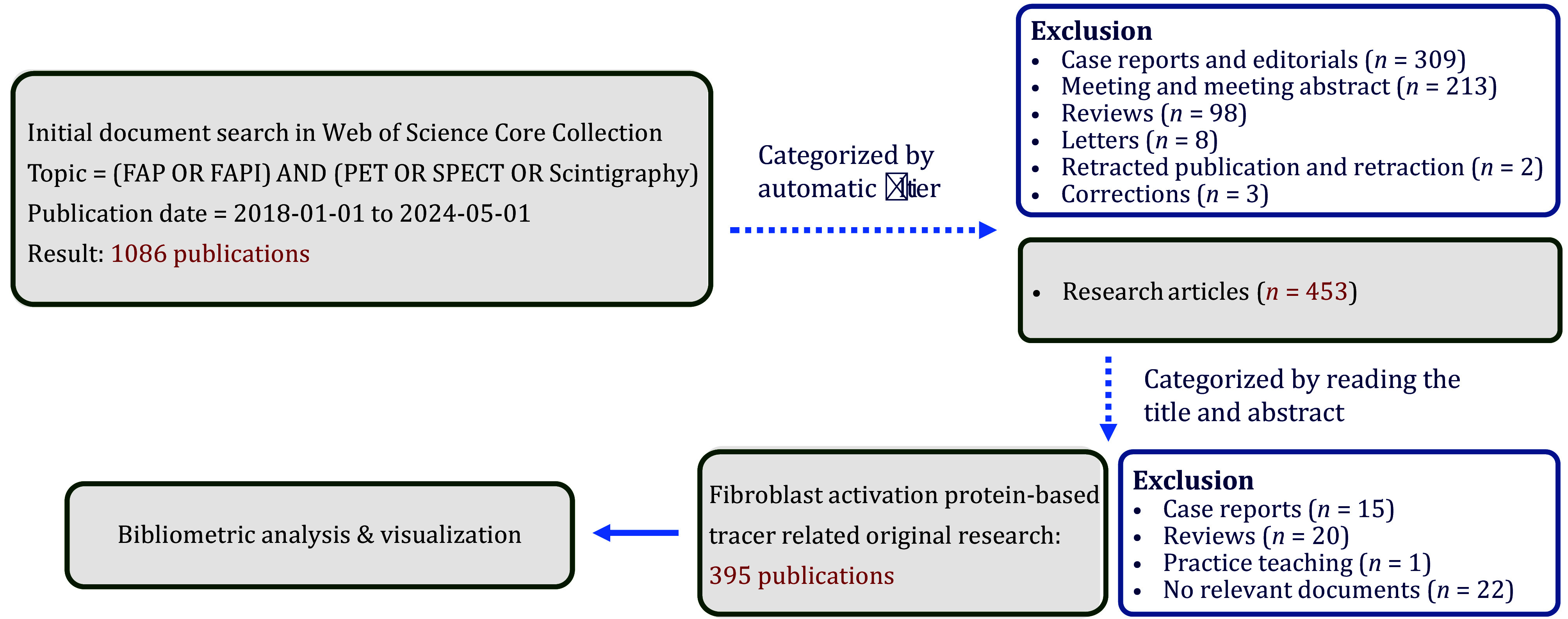
Flowchart of the retrieval of original research on radiolabeled FAPIs

After the search and inclusion process for radiolabeled FAPI, 395 original studies were included (Fig. 7), primarily focusing on diagnostic PET imaging (81.8%, 323/395). The number of studies has been increasing annually. Studies of common FAPI-based tracers (55.2%, 218/395) have been gradually increasing, but studies of new FAPI-based tracers (26.6%, 105/395) have been increasing by an even greater margin. Additionally, research on FAP-targeted radioligand therapy has been gradually increasing since 2021 (Table 1). Research methodologies encompassed comparisons between two PET tracers (50.6%, 200/395) (^68^Ga/^18^F-FAPI vs. ^18^F-FDG, ^68^Ga/^18^F-FAPI vs. other tracers or conventional imaging (CT, MRI, and ultrasonography), new FAPI variants vs. conventional FAPI-04/46), PET/CT with dual-tracer applications (1.5%, 6/395) (^68^Ga-FAPI + ^18^F-FDG), and single-tracer applications (46.8%, 185/395). Notably, publications involving head-to-head comparisons of radiolabeled FAPI and ^18^F-FDG PET imaging have surged since 2021 (Table 2).

**Table 1:** Basic information on the content of studies on radiolabeled FAPIs

	Number of studies (%)	2018	2019	2020	2021	2022	2023	2024
Total number of studies	395 (100.0%)	3 (0.8%)	5 (1.3%)	14 (3.5%)	50 (12.7%)	110 (27.8%)	160 (40.5%)	53 (13.4%)
Imaging	323 (81.8%)	1 (0.3%)	3 (0.9%)	14 (4.3%)	46 (14.2%)	91 (28.2%)	128 (39.6%)	40 (12.4%)
^a^Common FAPI-based tracers	218 (55.2%)		3 (1.4%)	8 (3.7%)	39 (17.9%)	67 (30.7%)	77 (35.3%)	24 (11.0%)
^b^New FAPI-based tracers	105 (26.6%)	1 (1.0%)		6 (5.7%)	7 (6.7%)	24 (22.9%)	51 (48.6%)	16 (15.2%)
Radioligand therapy	15 (3.8%)				3 (20.0%)	6 (40.0%)	5 (33.3%)	1 (6.7%)
Imaging & Radioligand therapy	25 (6.3%)	2 (8.0%)	2 (8.0%)			6 (24.0%)	9 (36.0%)	6 (24.0%)
Imaging techniques (Recon-algorithm, Dynamic imaging, Dual-tracer, Acquisition time, Tracer dose, Administration route)	16 (4.1%)					3 (18.8%)	11 (68.8%)	2 (12.5%)
Synthesis of FAPI-based radioactive molecular	16 (4.1%)				1 (6.3%)	4 (25.0%)	7 (43.8%)	4 (25.0%)
^68^Ga-FAPI-46	2 (0.5%)					1 (50.0%)		1 (50.0%)
New FAPI-based tracers	14 (3.5%)				1 (7.1%)	3 (21.4%)	7 (50.0%)	3 (21.4%)
^a^Common FAPI-based tracers: ^68^Ga-FAPI-02/04/46/74 ^b^New FAPI-based tracers: ^68^Ga-labeled FAPI-based tracers (^68^Ga-mhLL1, ^68^Ga-Alb-FAPtp-01, ^68^Ga-AV01017/AV01030/AV01038/AV02053/AV02070, ^68^Ga-DATA^5m^.SA.FAPi, ^68^Ga-DOTA-2P(FAPI)2, ^68^Ga-DOTA-FL, ^68^Ga-DOTA-GPFAPI-04, ^68^Ga-DOTA-Siglec-9, ^68^Ga-DOTA.SA.FAPi, ^68^Ga-FAP-2286, ^68^Ga-FAP-2286-ICG. ^68^Ga-FAPI-PSMA. ^68^Ga-FAPI-RGD, ^68^Ga-HBED-CC-FAPI, ^68^Ga-LNC1007/LNC1013, ^68^Ga-OncoFAP,^68^Ga-PNT6555, ^68^Ga-SB04028/SB03045/SB03058); ^18^F-labeled FAPI-based tracers (^18^F-1/2/3/6/12/13, ^18^F-FAP-2286, ^18^F-FAPI-04/42/74, ^18^F-FAPI-MB, ^18^F-FAPI-RGD, ^18^F-FAPT, ^18^F-FAPTG, ^18^F-FGlc-FAPI, ^18^F-ND-bisFAPI, ^18^F-NOTA-DD-FAPI, ^18^F-NOTA-PD-FAPI, ^18^F-P-FAPI, ^18^F-PSMA-FAPI-01/02); ^99m^Tc-labeled FAPI-based tracers (^99m^Tc-[Tc-(CN-C5-FAPI)6]^+^/[Tc-(CN-PEG4-FAPI)6]^+^, ^99m^Tc-DP-FAPI, ^99m^Tc-FAPI-04/34, ^99m^Tc-FL-L3, ^99m^Tc-tricine(2)-HYNIC-Glc-FAPT, ^99m^Tc-tricine/EDDA-HYNIC-Glc-FAPT); Other nuclide-labeled FAPI-based tracers (^64^Cu-TMs, ^64^Cu-FP-L1/L2, ^64^Cu-FAPI-04, ^11^C-FAPI, ^111^In-28H1, ^89^Zr-B12 IgG, ^89^Zr-Df-Bz-F19)								

**Table 2:** Methodology of studies on radiolabeled FAPIs

	Number of studies (%)	2018	2019	2020	2021	2022	2023	2024
Comparison of imaging modality	200 (50.6%)	1 (0.5%)	2 (1.0%)	5 (2.5%)	29 (14.5%)	58 (29.0%)	79 (39.5%)	26 (13.0%)
Common FAPI-based radioactive molecular	130 (32.9%)	1 (0.8%)	2 (1.5%)	3 (2.3%)	25 (19.2%)	43 (33.1%)	42 (32.3%)	14 (10.8%)
^68^Ga-FAPI vs ^18^F-FDG	110 (27.8%)	1 (0.9%)	1 (0.9%)	1 (0.9%)	21 (19.1%)	37 (33.6%)	39 (35.5%)	10 (9.1%)
^68^Ga-FAPI vs ^18^F-FDG vs CI	6 (1.5%)				1 (16.7%)	2 (33.3%)	1 (16.7%)	2 (33.3%)
^68^Ga-FAPI vs ^18^F-FDG vs ^99m^Tc-MDP	1 (0.3%)						1 (100.0%)	
^68^Ga-FAPI vs ^99m^Tc-MDP	1 (0.3%)						1 (100.0%)	
^68^Ga-FAPI vs ^68^Ga-PSMA	1 (0.3%)					1 (100.0%)		
^68^Ga-FAPI vs CI	7 (1.8%)			2 (28.6%)	3 (42.9%)			2 (28.6%)
^68^Ga-FAPI vs Cardiac-MRI vs Perfusion-SPECT	1 (0.3%)					1 (100.0%)		
New FAPI-based radioactive molecular	70 (17.7%)			2 (2.9%)	4 (5.7%)	15 (21.4%)	37 (52.9%)	12 (17.1%)
^a^FAPI vs ^18^F-FDG	32 (8.1%)				3 (9.4%)	6 (18.8%)	18 (56.3%)	5 (15.6%)
^b^FAPI vs ^18^F-FDG vs CI	1 (0.3%)							1 (100.0%)
^c^FAPI vs FAPI vs ^18^F-FDG	6 (1.5%)						6 (100.0%)	
^d^FAPI vs ^18^F-FDG vs ^68^Ga-PSMA	1 (0.3%)							1 (100.0%)
^e^FAPI vs FAPI	27 (6.8%)			2 (7.4%)	1 (3.7%)	9 (33.3%)	10 (37.0%)	5 (18.5%)
^f^FAPI vs ^68^Ga-DOTANOC	1 (0.3%)						1 (100.0%)	
^g^FAPI vs CI	2 (0.5%)						2 (100.0%)	
Dual tracer applications (^68^Ga-FAPI-04/46 & ^18^F-FDG)	6 (1.5%)					2 (33.3%)	2 (33.3%)	2 (33.3%)
Single tracer applications	185 (46.8%)	2 (1.1%)	3 (1.6%)	9 (4.9%)	20 (10.8%)	49 (26.5%)	78 (42.2%)	24 (13.0%)
CI: conventional imaging (including CT, MRI, and ultrasonography); Common FAPI-based radioactive molecular: ^68^Ga-FAPI-02/04/46/74; ^a^FAPI: ^68^Ga-DOTA.SA.FAPi, ^68^Ga/^177^Lu-DOTAGA.(SA.FAPi)2, ^68^Ga-FAPI-RGD, ^18^F-FAPI-04, ^18^F-FAPI-42, ^18^F-FAPI-74; ^b^FAPI: ^18^F-FAPI; ^c^FAPI: ^68^Ga-FAP-2286, ^68^Ga-FAPI-RGD, ^68^Ga-LNC1007, ^68^Ga/^177^Lu-(FAPI-04)2, ^99m^Tc-HYNIC-FAPI-04; ^d^FAPI: ^68^Ga-LNC1007; ^e^FAPI: ^68^Ga-HBED-CC-FAPI, ^68^Ga-DOTA-GPFAPI-04, ^68^Ga-AV02053/-AV02070, ^68^Ga-AV01017/-AV01030/-AV01038, ^68^Ga-Alb-FAPtp-01, ^68^Ga-8-1/-3-3, ^68^Ga/^177^Lu-DOTA-2P(FAPI)2, ^68^Ga/^177^Lu-FSDD0I/-FSDD1I/-FSDD3I, ^68^Ga/111In/^177^Lu-FAP-2286, ^18^F-AlF-PSMA-FAPI-01/-02, ^18^F-AlF-P-FAPI, ^18^F-AlF-FAPT, ^18^F-FAPI-42, ^18^F-FAPTG, ^18^F-FGlc-FAPI, ^18^F/^177^Lu-21, ^18^F/^177^Lu-AlF-ND-bisFAPI, ^177^Lu-FAPI-46-F1D/-46-Ibu/-46-EB, 11C-RJ1101/-RJ1102, ^99m^Tc-FAPI-34; ^f^FAPI: ^68^Ga-DOTA.SA.FAPi; ^g^FAPI: ^99m^Tc-FAPI; ^18^F-FAPI								

Radionuclides used to label FAPIs for imaging included ^11^C, ^18^F, ^68^Ga, ^111^In, ^99m^Tc, ^64^Cu, ^89^Zr, and ^86^Y. Predominantly, ^68^Ga (72.7%) and ^18^F (17.5%) were extensively employed. Studies on ^68^Ga-labeled FAPI-based radiotracers increased dramatically from 2021 to 2023, and those on ^18^F-labeled FAPI-related compounds rose in 2022 and 2023. The therapeutic radionuclide^ 177^Lu saw increased usage in FAP-targeted radioligand therapy since 2021, while short-ray therapeutic radionuclides like ^225^Ac were developed in 2020 (Table 3). PET/CT served as the primary imaging modality in both animal experiments and clinical evaluations (Table 4).

**Table 3:** Radionuclides used in radiolabeled FAPI studies

	Number of studies (%)	2018	2019	2020	2021	2022	2023	2024
Imaging application								
^68^Ga	287 (72.7%)	2 (0.7%)	5 (1.7%)	8 (2.8%)	44 (15.3%)	82 (28.6%)	110 (38.3%)	36 (12.5%)
^18^F	69 (17.5%)			1 (1.4%)	3 (4.3%)	16 (23.2%)	36 (52.2%)	13 (18.8%)
^99m^Tc	12 (3.0%)			2 (16.7%)		4 (33.3%)	5 (41.7%)	1 (8.3%)
^86^Y	3 (0.8%)					3 (100.0%)		
^64^Cu	5 (1.3%)			1 (20.0%)		1 (20.0%)	2 (40.0%)	1 (20.0%)
^111^In	1 (0.3%)	1 (100.0%)						
^11^C	1 (0.3%)					1 (100.0%)		
^89^Zr	4 (1.0%)					2 (50.0%)	2 (50.0%)	
Radioligand therapy application								
^177^Lu	34 (8.6%)		2 (5.9%)		2 (5.9%)	11 (32.4%)	13 (38.2%)	6 (17.6%)
^90^Y	4 (1.0%)	1 (25.0%)				2 (50.0%)	1 (25.0%)	
^225^Ac	5 (1.3%)			1 (20.0%)		1 (20.0%)	2 (40.0%)	1 (20.0%)
^131^I	1 (0.3%)							1 (100.0%)

**Table 4:** Detection instruments used in radiolabeled FAPI studies

	Number of studies (%)	2018	2019	2020	2021	2022	2023	2024
Animal experiment								
Micro-PET OR micro-PET/CT	91 (23.0%)	2 (2.2%)	3 (3.3%)	3 (3.3%)	5 (5.5%)	25 (27.5%)	33 (36.3%)	20 (22.0%)
Micro-PET/MRI	2 (0.5%)		1 (50.0%)			1 (50.0%)		
Micro-SPECT OR micro-SPECT/CT	25 (6.3%)	1 (4.0%)			1 (4.0%)	7 (28.0%)	12 (48.0%)	4 (16.0%)
Clinical application								
PET/CT	289 (73.2%)	2 (0.7%)	3 (1.0%)	9 (3.1%)	42 (14.5%)	80 (27.7%)	118 (40.8%)	35 (12.1%)
PET/MRI	24 (6.1%)				2 (8.3%)	11 (45.8%)	7 (29.2%)	4 (16.7%)
SPECT or SPECT/CT or Scintigraphy	16 (4.1%)	1 (6.3%)		2 (12.5%)	2 (12.5%)	5 (31.3%)	4 (25.0%)	2 (12.5%)

The primary application of radiolabeled FAPIs was in cancer diagnosis (particularly for gastrointestinal malignancies), including pancreatic cancer (18.2%, 72/395), colorectal cancer (13.9%, 55/395), gastric cancer (11.4%, 45/395), liver and bile duct cancer (12.9%, 51/395), breast cancer (13.2%, 52/395), and lung cancer (12.2%, 48/395). Research pertaining to these malignancies was published primarily between 2021 and 2023. From 2021 onwards, the number of studies on inflammatory and autoimmune diseases gradually increased (Table 5). Notably, ^68^Ga-FAPI showed no advantages over ^18^F-FDG in diagnosing multiple myeloma (MM), lymphoma, certain head and neck malignancies, and IgG4-related lymphadenopathy (supplementary Table S4).

**Table 5:** Main diseases covered by radiolabeled FAPI studies

Types of disease	Number of studies (%)	2018	2019	2020	2021	2022	2023	2024
Lung cancer	48 (12.2%)	1 (2.1%)	2 (4.2%)	1 (2.1%)	9 (18.8%)	18 (37.5%)	13 (27.1%)	4 (8.3%)
Brain tumor (Gliomas & other)	16 (4.1%)		1 (6.3%)	4 (25.0%)	2 (12.5%)	5 (31.3%)	4 (25.0%)	
Digestive system tumors								
Pancreatic cancer	72 (18.2%)	1 (1.4%)	3 (4.2%)	4 (5.6%)	9 (12.5%)	21 (29.2%)	29 (40.3%)	5 (6.9%)
Colorectal cancer	55 (13.9%)		3 (5.5%)	2 (3.6%)	7 (12.7%)	17 (30.9%)	18 (32.7%)	8 (14.5%)
Gastric cancer	45 (11.4%)		1 (2.2%)	2 (4.4%)	5 (11.1%)	16 (35.6%)	17 (37.8%)	4 (8.9%)
Hepatobiliary cancer (liver & bile duct)	51 (12.9%)		2 (3.9%)	1 (2.0%)	9 (17.6%)	16 (31.4%)	18 (35.3%)	5 (9.8%)
Esophageal & Gastroesophageal junction cancer	33 (8.4%)		2 (6.1%)	2 (6.1%)	5 (15.2%)	9 (27.3%)	12 (36.4%)	3 (9.1%)
Gynecological cancer								
Breast cancer	52 (13.2%)	2 (3.8%)	3 (5.8%)	1 (1.9%)	11 (21.2%)	13 (25.0%)	18 (34.6%)	4 (7.7%)
Ovarian cancer	29 (7.3%)		3 (10.3%)	2 (6.9%)	8 (27.6%)	2 (6.9%)	11 (37.9%)	3 (10.3%)
Uterus cancer (cervical cancer, endometrial cancer)	17 (4.3%)		2 (11.8%)	1 (5.9%)	3 (17.6%)	4 (23.5%)	7 (41.2%)	
Urinary system tumors								
Prostate cancer	21 (5.3%)		2 (9.5%)	1 (4.8%)	3 (14.3%)	6 (28.6%)	8 (38.1%)	1 (4.8%)
Renal cancer	17 (4.3%)		2 (11.8%)		2 (11.8%)	6 (35.3%)	4 (23.5%)	3 (17.6%)
Urothelial-Bladder Cancer	10 (2.5%)				1 (10.0%)	5 (50.0%)	3 (30.0%)	1 (10.0%)
Head and neck cancer								
Thyroid cancer	22 (5.6%)		2 (9.1%)		5 (22.7%)	4 (18.2%)	9 (40.9%)	2 (9.1%)
Oral cancer	18 (4.6%)		1 (5.6%)	1 (5.6%)	4 (22.2%)	9 (50.0%)	2 (11.1%)	1 (5.6%)
Nasopharyngeal cancer	21 (5.3%)			1 (4.8%)	5 (23.8%)	11 (52.4%)	2 (9.5%)	2 (9.5%)
Other cancer								
Sarcoma	25 (6.3%)		2 (8.0%)	1 (4.0%)	3 (12.0%)	10 (40.0%)	6 (24.0%)	3 (12.0%)
Lymphoma	15 (3.8%)				3 (20.0%)	6 (40.0%)	5 (33.3%)	1 (6.7%)
Multiple Myeloma	4 (1.0%)				1 (25.0%)	2 (50.0%)	1 (25.0%)	
Metastases (Liver, bone, peritoneal, lymph node)	9 (2.3%)				2 (22.2%)		5 (55.6%)	2 (22.2%)
Benign disease								
Cardiac vascular disease	31 (7.8%)		1 (3.2%)		4 (12.9%)	7 (22.6%)	16 (51.6%)	3 (9.7%)
Acute / Chronic organic disease & Fibrosis	42 (10.6%)			1 (2.4%)	6 (14.3%)	12 (28.6%)	16 (38.1%)	6 (14.3%)
Joint Bone disease	17 (4.3%)	1 (5.9%)			2 (11.8%)	8 (47.1%)	4 (23.5%)	2 (11.8%)

## DISCUSSION

Since 1990, researchers have proposed using FAP antibodies as radioisotope-labeled ligands or as therapeutic agents in combination with toxic chemotherapeutic substances for cancer therapy (Garin-Chesa *et al.* 1990). However, the performance of modern antitumor drugs utilizing FAP vaccines, FAP antibodies (*e*.*g*., Sibrotuzumab), and FAP inhibitors remains unsatisfactory (Kelly *et al.* 2012; Liu *et al.* 2023; Xu *et al.* 2017). This is the first study to perform a thorough and comprehensive visual analysis of FAP-related research using a bibliometric approach. Unlike the previous study by van den Hoven *et al*., which solely focused on the use of radionuclide-labeled FAPI in oncological and non-oncological diseases (van den Hoven *et al.* 2023), our study encompasses research on the molecular mechanisms of FAP, FAP-related drugs and biomarkers, as well as the development and clinical evaluation of FAPI-based radiotracers. Our study highlights the most relevant and important findings in FAP development and underscores the need for an in-depth review of emergent FAP studies in the field of medicine.

Of the 53 countries publishing FAP research, China led in the number of publications, yet its global citation count and international collaboration efforts were limited, indicating conservative research practices. Germany, with the highest citations, showcased its authority and social influence in FAPI-related radiopharmaceuticals. Despite global interest in FAP-related research, enhanced collaborations are needed, especially among national centers other than the German center, to advance research in this field.

Half of the top ten most-cited articles (5/10) in the field of FAP-related studies investigated radiolabeled FAPIs (preclinical experiments and clinical investigations), highlighting the broad recognition of radiolabeled FAPIs. The remaining (5/10) highly cited articles primarily focused on FAP-positive immunotherapies and using FAP as a biomarker (supplementary Table S2). Analyzing journal impact in the FAP-targeted theranostics field aids researchers in choosing suitable journals. EJNMMI had the most publications, predominantly featuring FAPI-related clinical investigations (73.1%), which is vital for exploring clinical indications in FAPI PET imaging. Both EJNMMI (36.5%) and JNM (36.2%) emphasized developing novel FAPI-based radiotracers and therapeutic radiopharmaceuticals, conducting preliminary clinical trials (supplementary Table S4). The *Clinical Nuclear Medicine*, a Zone-1 journal, presented numerous case reports detailing ^68^Ga/^18^F-FAPIs manifestations in rare diseases, valuable reference for future disease diagnosis studies. The *Cancers*, another Zone-1 journal, emphasized FAP expression in malignant tumors and non-radioactive drug treatment; over half of these articles were reviews, including some on radiolabeled FAPIs.

The surge in articles since 2018 is primarily due to radiolabeled FAPI development and clinical application. Most studies concentrated on disease diagnosis, but attention has gradually shifted to tumor stroma-targeted radioligand therapy since 2022. Recent research delved into novel FAPI variants, assessing their uptake, retention, and dosimetry in tumors and normal organs, crucial for therapeutic effectiveness in FAP-targeted radioligand therapy. Novel radiotracers like ^68^Ga-DOTA-2P(FAPI)_2_, ^68^Ga-DOTAGA.(SA.FAPi)_2_, and ^68^Ga-DOTAGA.Glu.(FAPi)_2_, exhibited superior tumor uptake and retention than common ^68^Ga-FAPI-04/46 (Ballal *et al.* 2022; Martin *et al.* 2023; Zhao *et al.* 2022). Researchers developed bispecific heterodimer radiotracers, like ^68^Ga-FAPI-RGD and ^68^Ga/^18^F-labeled FAPI-PSMA, demonstrating high tumor uptake and favorable *in vivo* pharmacokinetics (Wang *et al.* 2023; Zang *et al.* 2022).

Due to ^18^F-FDG’s limitations in certain cancers, most studies compared the diagnostic accuracies of FAPI-based radiotracers and ^18^F-FDG. Clinical studies have confirmed that ^68^Ga-labeled FAPI exhibits higher tumor uptake and diagnostic accuracy than ^18^F-FDG, especially in gastrointestinal cancers, notably gastric cancer (Li *et al.* 2023; Miao *et al.* 2023). FAPI-based radiotracers prove more sensitive than ^18^F-FDG in identifying recurrent disease, metastatic lymph nodes, brain metastases, liver metastases, and peritoneal metastases. In inflammatory disorders like rheumatoid arthritis, ^18^F-FAPI uptake was significantly greater in the early phase of inflammation than ^18^F-FDG uptake (Ge *et al.* 2022). However, in certain diseases (*e*.*g*., MM, lymphoma, and IgG4-related lymphadenopathy), FAPI-based radiotracers may be comparable or even inferior to ^18^F-FDG. A few studies reported that ^18^F-FDG performed better than ^68^Ga-FAPI in detecting metastatic lymph nodes in head and neck cancer; however, no histopathological analysis has been performed in FDG+/FAPI– lymph nodes. Owing to the high specificity of FAPI PET in diagnosing lymph node metastases, lymph nodes with increased ^18^F-FDG uptake may be reactive instead of metastatic (Demmert *et al.* 2023). Novel FAPI-based radiotracers, like ^68^Ga-DOTA.SA.FAPI and ^18^F-FAPI-04/42/74, showed superior diagnostic performance than ^18^F-FDG, as shown in recent clinical trials, detecting primary and metastatic lesions in breast cancer, metastatic/recurrent gastrointestinal stromal tumors, renal cancer, and lung cancer (Ballal *et al.* 2023; Wang *et al.* 2021; Wu *et al.* 2022; Zang *et al.* 2022). The combined use of ^18^F-FDG and ^68^Ga-FAPI (dual tracer for PET/CT) improved diagnostic accuracy in esophageal, gastric, cervical, and appendiceal cancer (Miao *et al.* 2023; Roth *et al.* 2022).

^68^Ga was the most frequently used radionuclide in diagnostic imaging, followed by ^18^F. However, ^68^Ga is limited to production because it generally requires a ^68^Ge/^68^Ga generator, which yields only enough for 2–4 patients per session, making it unsuitable for large clinical centers. Its short half-life (68 min) adds impracticality for transport to remote medical facilities. Conversely, cyclotrons can generate substantial amounts of ^18^F with a longer half-life (110 min). Consequently, some research centers favored the ^18^F-AIF-labeled chelator ligand FAPI-74 (Giesel *et al.* 2021; Kesch *et al.* 2017). Due to the high cost of PET scanning, ^99m^Tc-labeled FAPI with SPECT imaging may become an equally popular low-cost alternative (Lindner *et al.* 2020).

Regarding therapeutic radionuclides, most research centers favored ^177^Lu, followed by ^90^Y. When labeled with FAPIs, both nuclides utilize β-rays to target the tumor’s double-stranded DNA, inducing apoptosis. ^90^Y, with a shorter half-life (64.1 hours vs. 6.7 days) and higher average energy (0.9 MeV vs. 0.14 MeV) than ^177^Lu (Banerjee *et al.* 2015; Kersting *et al.* 2023), may be better suited for FAPI ligands with shorter tumor retention (*e*.*g*., FAPI-46 and FAPI dimers) (Fendler *et al.* 2022; Lindner *et al.* 2018). Promising outcomes in tumor treatment were observed with long-life ^131^I-labeled FAPI-02 and FAPI-04 (Ma *et al.* 2021). Besides β-ray-emitting therapeutic agents, α-ray-emitting therapeutic agents like ^225^Ac-FAPI-04 effectively inhibited tumor growth in pancreatic cancer xenografts (Watabe *et al.* 2020).

Animal experiments and clinical trials often require PET/CT, PET/MR, or SPECT/CT. CT scans are ineffective for soft-tissue lesions due to low resolution, while MRI provides superior soft-tissue resolution. PET/MRI holds promise in compensating for PET/CT limitations, especially in detecting small pancreatic carcinomas, distinguishing between potential tumors, and identifying brain and small liver metastases (Yeh *et al.* 2018; Zhang *et al.* 2022b). Multi-sequence MRI aids in interpreting inflammatory and physiologic uptake of ^68^Ga-FAPI in the uterus (Qin *et al.* 2022; Zhang *et al.* 2022a). SPECT, a cost-effective alternative to PET, is crucial for assessing dosimetry with ^177^Lu-labeled FAPI variants (Jia *et al.* 2023; Lindner *et al.* 2020).

FAPIs, acting as non-specific tracers, might be taken up by both malignant tumors and inflammatory tissues, complicating lesion image interpretation. Additionally, FAP expression doesn’t significantly correlate with tumor metastasis or staging. Owing to the dynamic changes in the composition of the extracellular matrix and CAFs during tumor growth and metastasis, FAPI-based radiotracer uptake may be altered throughout the course of tumor metastasis (Ding *et al.* 2021). Furthermore, FAPI-based radiotracers may be less accurate than ^18^F-FDG for specific cancers, like lymphomas and multiple myeloma (Kashyap and Ravi Kumar 2023). Thus, FAPI-based radiotracers may serve better as complementary diagnostic and therapeutic assessment tools rather than a comprehensive alternative to traditional ^18^F-FDG-based approaches.

This study has some limitations. Relevant literature might be excluded if the word of interest doesn’t appear in the search field. Additionally, due to the adaptability of the visualization software, only the Web of Science database was used, overlooking certain publications. Ongoing research, publication delays, and fluctuating attributes in citations, publication numbers, and keyword frequency mean we only considered FAP-based research trends in oncology from January 1, 1990, to May 1, 2024. Future studies should incorporate the most recent information to further elucidate advances in this area.

## CONCLUSIONS

In summary, after more than 30 years of continuous research and development, FAP has not only been characterized as an effective biomarker in disease diagnosis and prognosis but also as a promising target for tumor therapy. A particularly important milestone was the invention of radiolabeled FAPIs as novel radiotracers in the PET imaging of various cancer types. Radiolabeled FAPI is the foremost discovery in FAP research and has shown promising results, although research is in its preliminary stages. Thus, future research should focus on identifying the current limitations of FAPIs to inform their development for clinical applications. The field of oncology is increasingly dedicated to the advancement of FAPI, including assessment of the specific indications for FAPI PET imaging, development of novel FAPI-based radiotracers, and FAP-targeted radioligand therapy in refractory cancers. Finally, our findings highlighted the scope for improved collaboration between different countries and research institutions to broaden the application of FAP in disease diagnosis and treatment.

## MATERIAL AND METHODS

### Search strategy and data collection

To ensure the accuracy and quality of the study, data were exclusively sourced from the Core Collection of Web of Science. We used the search formula Topic = “fibroblast activation protein” to retrieve all FAP-related literature. The publication date was limited from January 1, 1990, to May 1, 2024. In addition, we analyzed the content and trends in FAPI-based radiotracer research from 2018 to 2024. The inclusion criteria were original studies of FAP in the field of nuclear medicine and molecular imaging. Case reports, reviews, comments, and non-full text were excluded. Two authors (R.D. and C.H.) performed the literature search and screening process.

## Data visualization

We used the bibliometric package (RRID: SCR_023744) in R (version 4.3.1) to extract the data required for the bibliometric analysis, including publication year, country, author, author keywords, affiliation, reference citations, and journal data. R was used to plot the data for statistical analysis. In addition, we used VOSviewer (version 1.6.18) to plot a collaboration network between countries.

## Data analysis

We extracted key information from original research articles on radiolabeled FAPIs for data analysis, including study content, methodology, type of radionuclide, imaging modality, and disease type. In addition, for the comparative studies between ^18^F-FDG-PET and FAPI-PET, we paid special attention to the limitations that exist in some FAPI applications. Finally, we analyzed the research hotspots and trends based on the distribution of study characteristics throughout the years.

## Abbreviations

**Table:** 

FAP	Fibroblast-activation protein
FAPI	Fibroblast-activation protein inhibitor
FDG	Fluorodeoxyglucose
PET/CT	Positron emission tomography/computed
CAF	Cancer-associated fibroblast
TME	Tumor microenvironment
SPECT	Single photon emission computed tomography
NIR	Near-infrared
PET/MRI	Positron emission tomography/magnetic
MM	Multiple myeloma resonance imaging

## Conflict of interest

Dan Ruan, Simin Wu, Xuehua Lin, Liang Zhao, Jiayu Cai, Weizhi Xu, Yizhen Pang, Qiang Xie, Xiaobo Qu and Haojun Chen declare that they have no conflict of interest.
